# Outcome of rectal cancer surgery in obese and nonobese patients: a meta-analysis

**DOI:** 10.1186/s12957-016-0775-y

**Published:** 2016-01-25

**Authors:** Yuan Qiu, Quanxing Liu, Guoqing Chen, Wensheng Wang, Ke Peng, Weidong Xiao, Hua Yang

**Affiliations:** 1 Department of General Surgery of Xinqiao Hospital, the Third Military Medical University, Shapingba, 400037 Chongqing, China; 2 Department of Thoracic Surgery of Xinqiao Hospital, the Third Military Medical University, Shapingba, 400037 Chongqing, China

**Keywords:** Obesity, BMI, Rectal surgery, Rectal cancer, Morbidity

## Abstract

**Background:**

The escalating global epidemic of obesity is of worldwide concern because of its association with serious negative effects on health. The technical difficulty of rectal cancer surgery is exacerbated in obese patients, which may compromise outcomes. High-quality, relevant evidence is limited. This meta-analysis aims to assess the outcomes of rectal cancer surgery in obese and nonobese patients.

**Methods:**

The electronic databases Pubmed, Medline, Embase, Web of Science, and the Cochrane Library were used to search for articles that evaluated the outcomes of rectal cancer surgery in obese and nonobese patients. Fixed-effects and random-effects models were used to calculate the combined overall effect sizes of pooled data. Data are presented as odds ratios (OR) or weighted mean differences (WMD) with 95 % confidence intervals (CIs).

**Results:**

Ten appropriate observational studies were identified from 290 published articles. In the obese group, conversion rates (OR 2.78; 95 % CI 1.67–4.61), overall morbidity (OR 1.36; 95 % CI 1.25–1.47), anastomotic leak (OR 3.94; 95 % CI 1.88–8.24), wound infection (OR 2.22; 95 % CI 1.47, 3.36), and pulmonary events (OR 2.10; 95 % CI 1.18, 3.74) were all significantly increased. For pathological results, no statistical differences in the number of harvested lymph nodes and the positive margin were noted between the two groups.

**Conclusions:**

Based on a meta-analysis, obesity increases the conversion rate and postoperative morbidity of rectal cancer surgery but does not influence pathological results.

## Background

Over the past few decades, obesity has become a global epidemic. As a result, there are many obese patients in most clinic practices, and in the USA, they constitute a third of all adult patients [1]. Body mass index (BMI), a good indicator of total body fat, is associated with adverse outcomes including metabolic, cardiovascular, musculoskeletal, neurologic, respiratory, and gastrointestinal disturbances, which can adversely affect surgical outcomes [2]. Furthermore, perhaps because of its etiological association with colorectal cancer, obesity is common in patients with this disease [3]. However, whether a higher BMI can compromise surgical outcomes in patients with rectal cancer is unclear.

Rectal cancer surgery presents unique challenges; the limited size of the pelvic cavity, necessity for extensive yet precise dissection, and the proximity of other major anatomic structures, all contribute to a complication rate that has been reported at 30 to 50 %, substantially higher than the 10 to 12 % cited for general surgery procedures overall [4–6]. High BMI may be assumed to increase the technical difficulty of rectal cancer surgery. The limiting pelvic cavity can be further restricted by visceral adiposity, a bulky mesentery can reduce mobility, and a thickened abdominal wall can complicate ostomy formation [6]. Although many surgeons acknowledge the challenges of rectal cancer surgery in the obese, the exact impact of obesity on outcomes for rectal cancer surgery remains controversial. Some studies suggested that patients with higher BMI had significantly more conversions to an open procedure, more postoperative complications, and a higher morbidity rate after rectal cancer resection, whereas others yielded conflicting results [7, 8]. In addition, whether higher BMI would have an adverse impact on pathological results is still debated. In this study, we conducted a comprehensive meta-analysis of published studies to derive a summary estimate of the associations between obesity and outcomes for rectal cancer surgery.

## Methods

### Study selection

This meta-analysis was conducted and reported according to the MOOSE guidelines [9]. Our search strategy was performed in the databases of PubMed/Medline, Embase, Web of Science, and the Cochrane Library until December 2014 to identify the eligible observational studies that evaluated the outcomes of rectal cancer surgery in obese and nonobese patients. Search items included “obesity” or “obese” or “body mass index (BMI)” and “rectal cancer” or “rectal carcinoma” or “laparoscopy” or “resection” or “rectal surgery.” Only studies that were conducted on humans and published in the English language were considered for inclusion. We then searched the reference lists of all relevant articles retrieved from the computerized database search to find other potentially relevant articles. When the same patient population was included in several publications, only the results from the most recent or most complete study were used in the present meta-analysis.

### Data extraction

Two of this study’s authors (Y. Qiu and Q.X. Liu) independently extracted the data from all eligible studies. If these two authors could not reach a consensus, disagreements were discussed and resolved by a third author (G.Q. Chen). The following variables were recorded: last name of the first author, publication year, country in which the study was performed, participant characteristics, sample size, study design, inclusion and exclusion criteria, and outcomes. Considering the observational study design of the included studies, the Newcastle-Ottawa Scale (NOS) was obtained to assess the methodological quality of the included studies. It assessed the patient selection, comparability of the study groups, and the outcomes. A maximum of 9 was scored for a study and the study with over 6 would be regarded as relative high-quality [10].

### Criteria for inclusion and exclusion

The following inclusion criteria were used for the present meta-analysis: (1) outcomes of rectal cancer surgery in obese and nonobese patients were compared; (2) BMI was used to determine obesity; and (3) at least one of the perioperative outcomes or pathological results (see below) and the standard deviation for the mean for continuous outcomes of interest were reported or could be calculated. Case reports, letters, editorials, comments, reviews, and abstracts with insufficient details to meet the inclusion criteria were excluded. Differences in classifications of obesity might affect homogeneity. All of the studies used BMI as a measure of obesity and nonobese. According to the recommendation of the WHO, the categorical definition of obesity used here was a BMI of ≥30 kg/m^2^, and BMI <30 kg/m^2^ was considered nonobesity [11].

### Clinical outcomes of surgery for rectal cancer

- ♦ Operative outcome: conversion rate.
- ♦ Postoperative outcomes: overall morbidity, ileus, wound infection, pulmonary events, deep venous thrombosis, anastomotic leak, intra-abdominal abscess, intra-abdominal hematoma, fistula, reoperation, postoperative mortality, cardiac complications, renal failure, sexual dysfunction, and bladder dysfunction.
- ♦ Pathological results: the positive margin and the number of harvested nodes.

### Statistical analysis

We analyzed dichotomous variables using an estimation of the odds ratios (OR) with a 95 % confidence interval (95 % CI) and continuous variables using a weighted mean difference (WMD) with a 95 % CI. The pooled effect was calculated using a fixed-effects or random-effects model. Statistical heterogeneity was evaluated through the use of *χ* ^2^ and *I* ^2^ statistics. We considered heterogeneity to be present if the *I* ^2^ statistic was >50 %. *P* < 0.05 was considered to be significant.

## Results

### Search results and study characteristics

**Fig. 1:**
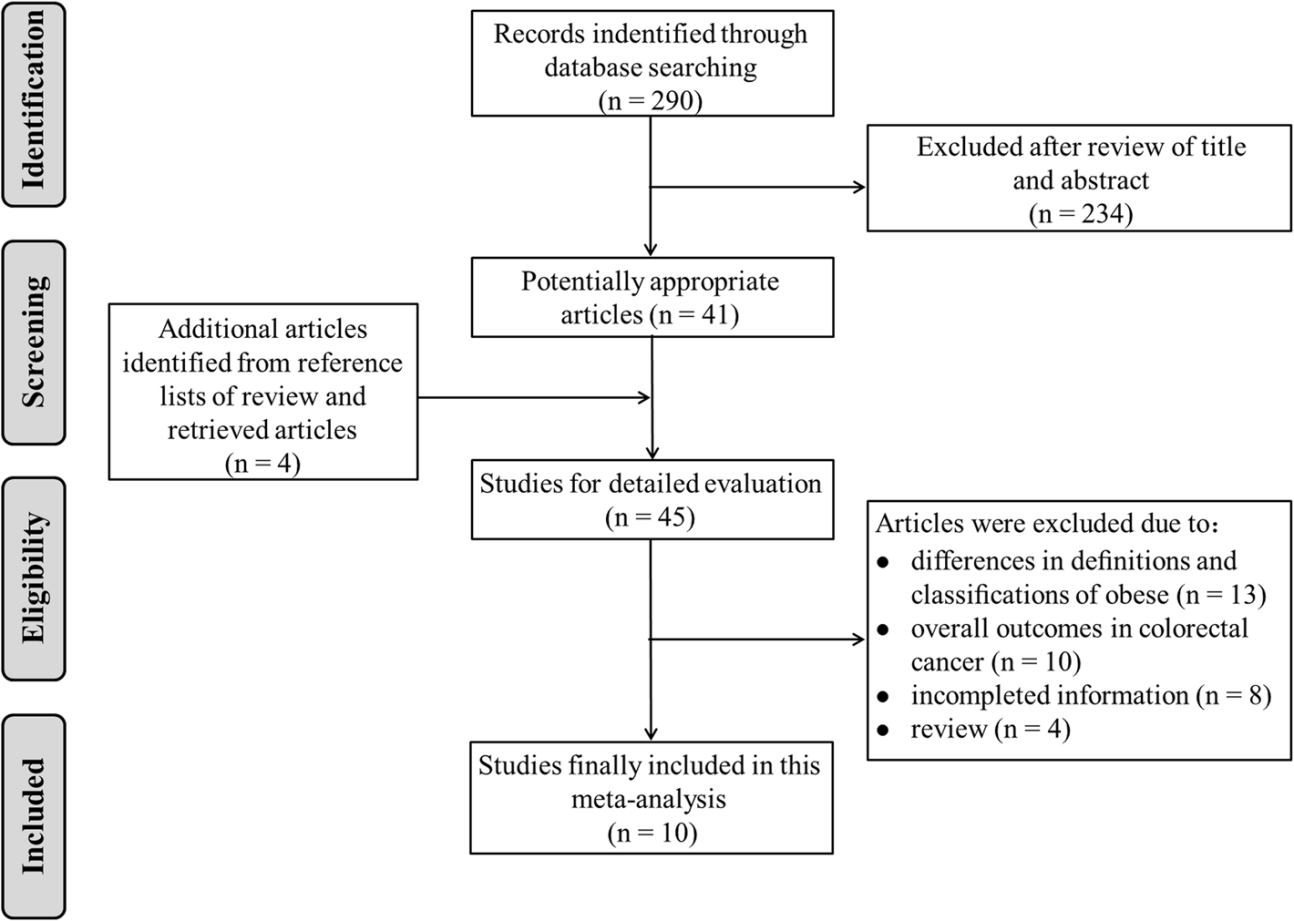
Flow diagram of studies identified, included, and excluded

A total of 290 articles were initially identified. The titles and abstracts were reviewed to exclude the irrelevant studies. We identified 45 potentially eligible articles that evaluated the impact of obesity on the outcomes of rectal cancer surgery. Finally, 10 studies complied with all inclusion criteria [4, 7, 8, 12–18]. Figure 1 provides a flow diagram of the search.

The characteristics of the included studies are shown in Table 1. A total of 14,489 patients (obese 3660, nonobese 10,829) from 10 studies were included. The sample size of each study varied from 26 to 11,995 patients. Of the included studies, six studies were conducted in the USA [4, 8, 13, 15, 16, 18], two in France [7, 14], one in Turkey [17], and one in Australia [12]. All of the studies used BMI as a measure of obesity and nonobesity; however, the cutoff points used to categorize BMI varied among the studies. Four studies split BMI into at least three tiers: (1) normal weight, BMI 18.5–24.9 or 20–24.9 or <25; (2) overweight, BMI 25–29.9; and (3) obese, BMI ≥ 30 [8, 13, 14, 17]. The remaining studies assessed BMI as obesity (BMI ≥ 30) versus nonobesity (BMI < 30). In these studies, patients in the two groups were matched for age, gender, American Society of Anesthesiologists (ASA) score, diagnosis, and surgical procedure performed. Moreover, according to the NOS, all the included studies belonged to a high-quality class (NOS score ≥ 6).

**Table 1:** Characteristics of the 10 selected studies included in the meta-analysis

Study	Year	Country	Study size	Group (OB/NOB)	NOS	Matching
Bokey et al. [12]	2014	Australia	255	95/160	8	1, 2, 4, 5
Smith et al. [13]	2014	USA	11,995	3050/8945	6	4, 5
Denost et al. [14]	2013	France	490	47/443	7	1, 3, 4, 5
Aytac et al. [15]	2013	USA	471	157/314	9	1–5
Clark et al. [16]	2013	USA	96	39/57	6	2, 4
Oyasiji et al. [8]	2012	USA	26	7/19	7	1, 3, 4,
Karahasanoglu et al. [17]	2011	Turkey	100	14/86	7	1, 2, 4, 5
Ballian et al. [4]	2010	USA	254	68/186	9	1–5
Chern et al. [18]	2010	USA	592	159/433	8	1, 2, 4, 5
Bege et al. [7]	2009	France	210	24/186	6	1, 2, 4, 5

*NOS* Newcastle-Ottawa scale, *OB* obese, *NOB* nonobese

1 = age; 2 = gender; 3 = American Society of Anesthesiologists score; 4 = diagnosis; 5 = surgical procedures

### Meta-analysis of the conversion rates

**Fig. 2:**
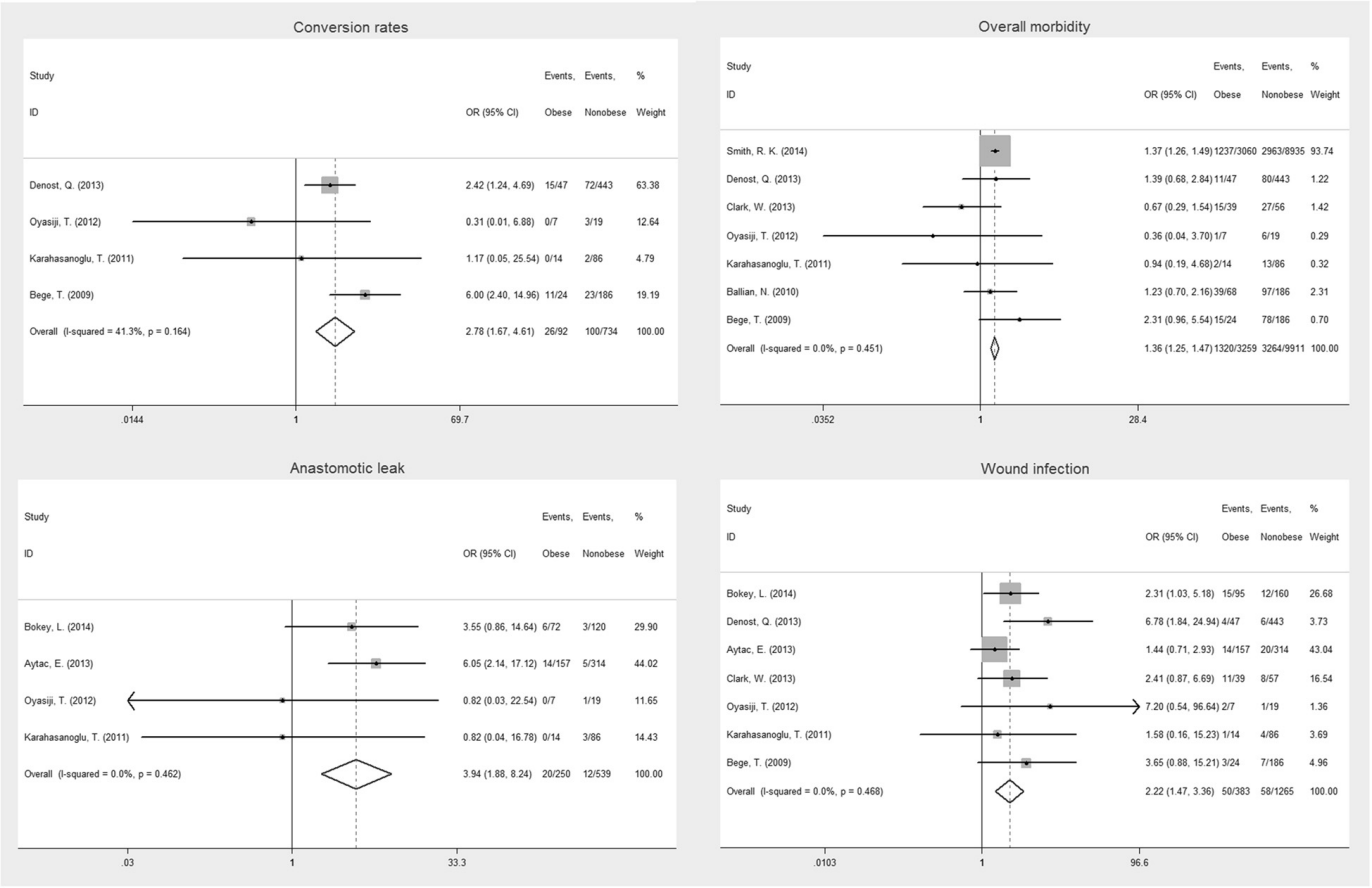
Forest plot displaying the results of the meta-analysis on conversion rates, overall morbidity, anastomotic leak, and wound infection

With respect to operative outcome, four studies reported the conversion rate. The conversion rate was significantly higher in obese patients than in nonobese patients (*P* < 0.001; Fig. 2).

### Meta-analysis of the postoperative outcomes

The main results of the meta-analysis are outlined in Table 2. Our estimated pooled OR clearly shows that obesity increased the risk of postoperative morbidity (*P* < 0.001; Fig. 2). Anastomotic leak is a major concern in obese patients because they may experience more intraoperative technical difficulties and comorbidities. The anastomotic leak rate was significantly higher in obese patients than in nonobese patients (*P* < 0.001; Fig. 2). In addition, wound infection (*P* < 0.001; Fig. 2) and pulmonary events (*P* = 0.012) were significantly higher in the obese group.

**Table 2:** Meta-analysis of the impact of obesity on outcomes after rectal cancer surgery

Outcome of interest	No. of studies	Events		OR/WMD	95 % CI	*P* value	*I* ^2^ (%)
		Obese (%)	Nonobese (%)				
Operative outcomes							
Conversion rates	4 [6, 7, 13, 16]	26/92 (28.3 %)	100/734 (13.6 %)	2.78	1.67, 4.61	<0.001	41.3
Postoperative outcomes							
Overall morbidity	7 [6, 7, 12, 13, 15–17]	1320/3259 (40.5 %)	3264/9911 (32.9 %)	1.36	1.25, 1.47	<0.001	0
Mortality	8 [6, 7, 11–14, 16, 17]	34/3472 (1.0 %)	109/10,329 (1.0 %)	0.95	0.65, 1.39	0.780	0
Ileus	4 [6, 7, 13, 14]	28/306 (9.2 %)	47/936 (5.0 %)	1.33	0.81, 2.20	0.260	49.2
Wound infection	7 [6, 7, 11, 13–16]	50/383 (13.1 %)	58/1265 (4.6 %)	2.22	1.47, 3.36	<0.001	0
Pulmonary events	5 [6, 7, 11, 14, 16]	23/297 (7.7 %)	28/765 (3.7 %)	2.10	1.18, 3.74	0.012	12.1
Deep venous thrombosis	2 [11, 14]	7/252 (2.8 %)	14/474 (3.0 %)	0.97	0.38, 2.43	0.941	0
Anastomotic leak	4 [7, 11, 14, 16]	20/250 (8.0 %)	12/539 (2.2 %)	3.94	1.88, 8.24	<0.001	0
Intraabdominal abscess	5 [7, 11, 13, 14, 16]	19/320 (5.9 %)	77/1022 (7.5 %)	1.10	0.65, 1.89	0.715	45.1
Intraabdominal haematoma	2 [11, 13]	5/142 (3.5 %)	18/603 (3.0 %)	0.65	0.24, 1.73	0.387	84
Fistula	3 [6, 11, 14]	5/276 (1.8 %)	27/660 (4.1 %)	1.15	0.40, 3.28	0.797	0
Reoperation	4 [6, 7, 11, 14]	14/283 (5.0 %)	46/679 (6.8 %)	0.79	0.43, 1.47	0.466	0
Cardiac complication	2 [11, 14]	19/252 (7.5 %)	29/474 (6.1 %)	1.17	0.63, 2.17	0.612	0
Renal failure	3 [7, 11, 14]	2/259 (0.8 %)	2/493 (0.4 %)	2.11	0.40, 11.03	0.378	0
Sexual dysfunction	2 [14, 16]	1/171 (0.6 %)	5/400 (1.3 %)	0.70	0.12, 4.25	0.699	0
Bladder dysfunction	2 [14, 16]	0/171 (0.0 %)	2/400 (0.5 %)	1.05	0.11, 10.27	0.965	0
Pathological results							
Positive margin	5 [7, 11, 14, 17, 18]	19/472 (4.0 %)	61/1079 (5.7 %)	0.71	0.42, 1.20	0.197	0
Number of harvested nodes	5 [6, 11, 15, 17, 18]			0.07	−0.69, 0.83	0.852	0

*OR* odds ratio, *WMD* weighted mean difference, *CI* confidence interval

However, there was no significant difference between the obese and nonobese groups for intra-abdominal abscess, deep venous thrombosis, intra-abdominal hematoma, fistula, reoperation, cardiac complication, renal failure, sexual dysfunction, and bladder dysfunction. Moreover, our estimated pooled OR shows that there was no significant difference between the two groups for postoperative mortality (*P* > 0.05).

### Meta-analysis of pathological results

No statistical difference was found between the two groups in terms of the number of harvested lymph nodes (*P* > 0.05). Pooled analysis for a positive margin showed a prevalence of 19 of 472 (4.0 %) in the obese group vs. 61 of 1079 (5.7 %) in the nonobese group, without reaching statistical significance (*P* > 0.05).

## Discussion

As the prevalence of being overweight and obesity has grown in Western countries, its potential effects on outcomes after abdominal surgery have received increasing attention. In obese patients, rectal resection for malignancy is clearly technically more difficult [19]. Most of the studies included in this analysis showed a significantly higher rate of conversion to laparotomy in an obese group (28.3 %) than in a nonobese group (13.6 %). These difficulties are primarily explained by problems of exposure (layering of intestinal loops, volume of mesorectum) and dissection difficulties due to the thickness of fat tissue, particularly in cases of visceral obesity. Moreover, the bulky mesentery is vulnerable to laceration and bleeding; therefore, using nontraumatic forceps to manipulate the bowel mesentery is both important and necessary [17]. Laceration due to traction of the mesentery may result in unacceptable bleeding, which makes the operation field chaotic. Unclear anatomy, intraoperative hemorrhage, intra-abdominal adhesions, and bowel perforation were reported to be the common causes for conversion in obese patients [20]. Moreover, other authors have found that male gender, BMI, and location of the rectal cancer were independent risk factors for conversion [21].

The results from our overall meta-analysis pointed to an association of obesity with increased morbidity in patients undergoing rectal cancer surgery. This observation is congruent with earlier studies that have associated obesity with greater technical difficulty and increased surgical morbidity in laparoscopic colorectal cancer resection [22]. In our study, obese group was proved to be a risk factor for wound infection. Wick et al. [23] also found that for patients undergoing colectomy, obesity increases the risk of developing a postoperative surgical site infection (SSI) by 60 %, with a cost in excess of $17,000. In fact, SSI can be difficult to prevent in an obese patient. This increased risk has been attributed to decreased oxygen tension in adipose tissue, tissue trauma, immune impairment, greater wound area, deficiencies in collagen synthesis, and prolonged surgery [24]. In addition, adiposity can affect the tissue concentrations of preoperative antibiotics [25]. Reducing infections by increasing antibiotic dosing and broadening the antibiotic spectrum during surgery may be possible.

Another important finding of the present analysis was that rate of anastomotic leak was significantly higher in the obese group (8.0 vs. 2.2 %, *P* < 0.001). Anastomotic leak is still the most dreaded surgical complication in colorectal surgery. This complication often results in reoperation and the need for a temporal or definitive stoma and consequently has a significant impact on the patient’s quality of life [26]. A retrospective analysis of 272 patients undergoing resection for rectal cancer found that a low-level anastomosis (≤5 cm below the anal verge) was an independent factor for the development of anastomotic leakage. In a second analysis of low anastomoses, obesity was the strongest risk factor for leakage; obese patients with a low anastomosis were more than twice as likely to experience a leak than were nonobese patients with a low anastomosis [27]. This finding may be explained by the fact that adiposity can affect the access of linear staplers to the distal rectum in a narrow pelvis, insertion of the stapler, or cephalad traction on the rectum [19, 28]. However, some multicenter studies have shown that surgeon-related variables could be risk factors for leakage [27, 29, 30]. Given these problems, surgeons must consider several factors that make anastomosis safe: bowel preparation, pelvic hemostasis, anastomotic tension, complete doughnuts, and intraoperative testing of the anastomosis.

Interestingly, we found that obese patients had higher incidence of pulmonary events when compared with nonobese patients. Under general anesthesia, body mass is an important determinant of vital capacity (VC), oxygenation, and respiratory mechanics, mainly affecting functional residual capacity (FRC) [31]. After a variety of surgical procedures, obese patients were found to have greater declines in VC and were more likely to have clinically significant pneumonia, atelectasis, and hypoxemia [24, 32]. Hence, the incidence of pulmonary complications was understandably more common in obese patients.

Obesity has been identified as an independent risk factor for postoperative ileus in some studies [33, 34]. A recent meta-analysis showed that obese patients had a significant higher rate of ileus after laparoscopic colectomy [22]. One might have expected to see an increase in the rate of ileus in the obese group in our study. However, results of this study showed that the incidence of the postoperative ileus did not differ significantly between the two groups. This result can in part be explained by the different operative scopes for colectomy and proctectomy. Alternatively, there was evidence of heterogeneity among the studies, most likely as a result of differences in receiving neoadjuvant chemotherapy among patients, tumor location in the rectum, and the learning curve for surgeons performing rectal surgery.

The presence of lymph node metastasis is important for predicting clinical outcomes in patients who have undergone radical surgery for rectal cancer and directing adjuvant therapy. Most previous reports of open and laparoscopic proctectomy have not shown an adverse effect for obesity on lymph node yield but have varied greatly in the number of nodes retrieved [35, 36]. This study showed no difference in the median number of lymph nodes harvested in each of the groups. Furthermore, a negative circumferential resection margin (CRM) was of crucial importance because radiotherapy could not compensate for a positive margin [37]. When the principles of total mesorectal excision are followed, CRM positivity should be minimized. We did not detect a higher rate of positive CRM in obese compared with nonobese rectal cancer patients in this meta-analysis. Therefore, the results suggest that obesity does not directly influence the pathological security.

The data analyzed in this meta-analysis suggest that rectal cancer surgery is more difficult in obese patients without affecting the pathological results. Nevertheless, these results should be interpreted cautiously because our analysis has several limitations. First, our analyses are of observational studies, and the inherent limitations of such studies may affect our findings. In the study selection process, we used the Western definition of obesity (BMI ≥ 30 kg/m^2^). Thus, some studies were excluded because they used different definition and classification of obesity. Second, differences in the study populations, assessment of covariates, and exposure variables may contribute to heterogeneity across the studies and thus affect the pooled estimates. Third, there is a lack of long-term data to quantitatively demonstrate the survival for patients with rectal cancer, which is clearly a controversial point when comparing prognosis in obese and nonobese patients [29]. Further analyses would have been of extreme interest, such as endoanal ultrasound and an anal sphincter assessment, low anterior resection syndrome score, and R0 achievement, but the lack of sufficient data on these topics did not permit us to analyze these factors further.

## Conclusions

The present study involving 14,489 subjects represents the first meta-analysis comparing the results of surgery for rectal cancer in obese and nonobese patients. This study shows that rectal cancer surgery is more difficult in obese patients, with an increase in the conversion to laparotomy and overall relevant morbidity and without an effect on the pathological results.

## References

[1] Kuczmarski RJ, Flegal KM, Campbell SM, Johnson CL (1994). Increasing prevalence of overweight among US adults. The national health and nutrition examination surveys, 1960 to 1991. JAMA.

[2] Blee TH, Belzer GE, Lambert PJ (2002). Obesity: is there an increase in perioperative complications in those undergoing elective colon and rectal resection for carcinoma?. Am Surg.

[3] Renehan AG, Tyson M, Egger M, Heller RF, Zwahlen M (2008). Body-mass index and incidence of cancer: a systematic review and meta-analysis of prospective observational studies. Lancet.

[4] Ballian N, Yamane B, Leverson G, Harms B, Heise CP, Foley EF (2010). Body mass index does not affect postoperative morbidity and oncologic outcomes of total mesorectal excision for rectal adenocarcinoma. Ann Surg Oncol.

[5] Mrak K, Eberl T, Fritz J, Tschmelitsch J (2012). Influence of body mass index on postoperative complications after rectal resection for carcinoma. South Med J.

[6] Hrabe JE, Sherman SK, Charlton ME, Cromwell JW, Byrn JC (2014). Effect of BMI on outcomes in proctectomy. Dis Colon Rectum.

[7] Bege T, Lelong B, Francon D, Turrini O, Guiramand J, Delpero JR (2009). Impact of obesity on short-term results of laparoscopic rectal cancer resection. Surg Endosc.

[8] Oyasiji T, Baldwin K, Katz SC, Espat NJ, Somasundar P (2012). Feasibility of purely laparoscopic resection of locally advanced rectal cancer in obese patients. World J Surg Oncol.

[9] Stroup DF, Berlin JA, Morton SC, Olkin I, Williamson GD, Rennie D (2000). Meta-analysis of observational studies in epidemiology: a proposal for reporting. Meta-analysis of observational studies in epidemiology (MOOSE) group. JAMA.

[10] Athanasiou T, Al-Ruzzeh S, Kumar P, Crossman MC, Amrani M, Pepper JR (2004). Off-pump myocardial revascularization is associated with less incidence of stroke in elderly patients. Ann Thorac Surg.

[11] Obesity: preventing and managing the global epidemic. Report of a WHO consultation. World Health Organ Tech Rep Ser. 2000;894:i-xii, 1–253. 11234459

[12] Bokey L, Chapuis PH, Dent OF. Impact of obesity on complications after resection for rectal cancer. Colorectal Dis. 2014 10.1111/codi.12726 25040856

[13] Smith RK, Broach RB, Hedrick TL, Mahmoud NN, Paulson EC (2014). Impact of BMI on postoperative outcomes in patients undergoing proctectomy for rectal cancer: a national surgical quality improvement program analysis. Dis Colon Rectum.

[14] Denost Q, Quintane L, Buscail E, Martenot M, Laurent C, Rullier E (2013). Short- and long-term impact of body mass index on laparoscopic rectal cancer surgery. Colorectal Dis.

[15] Aytac E, Lavery IC, Kalady MF, Kiran RP (2013). Impact of obesity on operation performed, complications, and long-term outcomes in terms of restoration of intestinal continuity for patients with mid and low rectal cancer. Dis Colon Rectum.

[16] Clark W, Siegel EM, Chen YA, Zhao X, Parsons CM, Hernandez JM (2013). Quantitative measures of visceral adiposity and body mass index in predicting rectal cancer outcomes after neoadjuvant chemoradiation. J Am Coll Surg.

[17] Karahasanoglu T, Hamzaoglu I, Baca B, Aytac E, Kirbiyik E (2011). Impact of increased body mass index on laparoscopic surgery for rectal cancer. Eur Surg Res.

[18] Chern H, Chou J, Donkor C, Shia J, Guillem JG, Nash GM (2010). Effects of obesity in rectal cancer surgery. J Am Coll Surg.

[19] Leroy J, Ananian P, Rubino F, Claudon B, Mutter D, Marescaux J (2005). The impact of obesity on technical feasibility and postoperative outcomes of laparoscopic left colectomy. Ann Surg.

[20] Tuech JJ, Regenet N, Hennekinne S, Pessaux P, Bergamaschi R, Arnaud JP (2001). Laparoscopic colectomy for sigmoid diverticulitis in obese and nonobese patients: a prospective comparative study. Surg Endosc.

[21] Thorpe H, Jayne DG, Guillou PJ, Quirke P, Copeland J, Brown JM (2008). Patient factors influencing conversion from laparoscopically assisted to open surgery for colorectal cancer. Br J Surg.

[22] Zhou Y, Wu L, Li X, Wu X, Li B (2012). Outcome of laparoscopic colorectal surgery in obese and nonobese patients: a meta-analysis. Surg Endosc.

[23] Wick EC, Hirose K, Shore AD, Clark JM, Gearhart SL, Efron J (2011). Surgical site infections and cost in obese patients undergoing colorectal surgery. Arch Surg.

[24] Gendall KA, Raniga S, Kennedy R, Frizelle FA (2007). The impact of obesity on outcome after major colorectal surgery. Dis Colon Rectum.

[25] Balentine CJ, Wilks J, Robinson C, Marshall C, Anaya D, Albo D (2010). Obesity increases wound complications in rectal cancer surgery. J Surg Res.

[26] Frasson M, Flor-Lorente B, Ramos Rodriguez JL, Granero-Castro P, Hervas D, Alvarez Rico MA, et al. Risk factors for anastomotic leak after colon resection for cancer: multivariate analysis and nomogram from a multicentric, prospective, national study with 3193 patients. Ann Surg. 2014. 10.1097/SLA.0000000000000973 25361221

[27] Rullier E, Laurent C, Garrelon JL, Michel P, Saric J, Parneix M (1998). Risk factors for anastomotic leakage after resection of rectal cancer. Br J Surg.

[28] Arezzo A, Passera R, Scozzari G, Verra M, Morino M (2013). Laparoscopy for rectal cancer reduces short-term mortality and morbidity: results of a systematic review and meta-analysis. Surg Endosc.

[29] You JF, Tang R, Changchien CR, Chen JS, You YT, Chiang JM (2009). Effect of body mass index on the outcome of patients with rectal cancer receiving curative anterior resection: disparity between the upper and lower rectum. Ann Surg.

[30] Martling A, Cedermark B, Johansson H, Rutqvist LE, Holm T (2002). The surgeon as a prognostic factor after the introduction of total mesorectal excision in the treatment of rectal cancer. Br J Surg.

[31] Pelosi P, Croci M, Ravagnan I, Tredici S, Pedoto A, Lissoni A (1998). The effects of body mass on lung volumes, respiratory mechanics, and gas exchange during general anesthesia. Anesth Analg.

[32] von Ungern-Sternberg BS, Regli A, Schneider MC, Kunz F, Reber A (2004). Effect of obesity and site of surgery on perioperative lung volumes. Br J Anaesth.

[33] Svatek RS, Fisher MB, Williams MB, Matin SF, Kamat AM, Grossman HB (2010). Age and body mass index are independent risk factors for the development of postoperative paralytic ileus after radical cystectomy. Urology.

[34] Artinyan A, Nunoo-Mensah JW, Balasubramaniam S, Gauderman J, Essani R, Gonzalez-Ruiz C (2008). Prolonged postoperative ileus-definition, risk factors, and predictors after surgery. World J Surg.

[35] Meyerhardt JA, Tepper JE, Niedzwiecki D, Hollis DR, McCollum AD, Brady D (2004). Impact of body mass index on outcomes and treatment-related toxicity in patients with stage II and III rectal cancer: findings from Intergroup Trial 0114. J Clin Oncol.

[36] Gorog D, Nagy P, Peter A, Perner F (2003). Influence of obesity on lymph node recovery from rectal resection specimens. Pathol Oncol Res.

[37] Marijnen CA, Nagtegaal ID, Kapiteijn E, Kranenbarg EK, Noordijk EM, van Krieken JH (2003). Radiotherapy does not compensate for positive resection margins in rectal cancer patients: report of a multicenter randomized trial. Int J Radiat Oncol Biol Phys.

